# New concepts for CML clonality

**DOI:** 10.18632/oncotarget.882

**Published:** 2013-02-17

**Authors:** Jamshid S. Khorashad, Michael W. Deininger, Thomas O'Hare

**Affiliations:** Huntsman Cancer Institute, The University of Utah, Salt Lake City, UT; Huntsman Cancer Institute; Division of Hematology and Hematologic Malignancies, The University of Utah, Salt Lake City, UT; Huntsman Cancer Institute; Division of Hematology and Hematologic Malignancies, The University of Utah, Salt Lake City, UT

**Figure 1:**
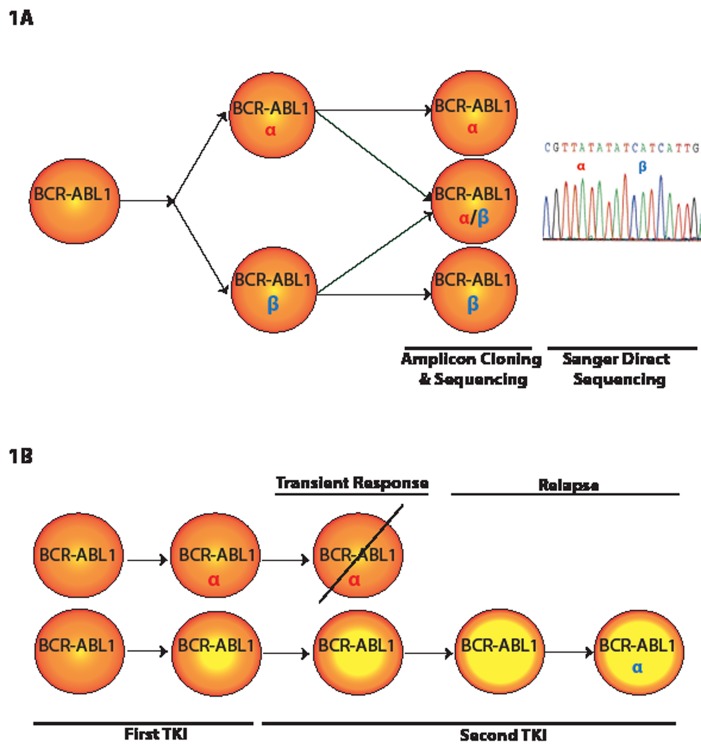
**A.** Model depicting two mutations in a compound mutant clone and also in parallel as two independent clones. A subclone of the mutant α clonal pool might acquire mutation β, resulting in co-existence of α and α/β pools. Independent and parallel acquisition of mutation β will add β to the pool of BCR-ABL1 positive cells. These events might also happen with β as the first mutation followed by acquisition of α. Cloning and sequencing of individual amplicons will discern mutants α and β from compound mutant α/β. Sanger direct sequencing identifies mutations α and β but does not establish whether they exist as a compound mutant. **B.** Model for emergence of a single mutation more than once during the course of disease. Resistance to the first-line TKI due to mutation α (red) in the *BCR-ABL1* kinase domain is addressed with a second-line TKI, resulting in a transient response and disappearance of α. A subclone lacking mutation α but carrying additional genomic abnormalities (represented with a higher yellow/orange ratio) may become resistant to first- and second-line TKIs. Acquisition of new α mutation (blue) might make this clone more resistant to the second-line TKI.

*BCR-ABL1* compound mutations, defined as ≥2 mutations within the same *BCR-ABL1* allele, can confer high-level resistance to imatinib and other ABL1 tyrosine kinase inhibitors (TKIs) in chronic myeloid leukemia (CML). Even the third generation ABL1 TKI, ponatinib, which is uniformly effective against BCR-ABL1 point mutants, remains vulnerable to certain BCR-ABL1 compound mutants [1, 7]. Therapy escape attributable to compound mutations may become more common with greater clinical use of ponatinib in refractory CML. To determine the frequency of compound mutations among CML patients on ABL1 TKI therapy, we examined cDNA samples (N=47) with clear Sanger direct sequencing evidence of two *BCR-ABL1* kinase domain mutations. Using an amplicon cloning and sequencing method, we confirmed that a high proportion of patients (70%; 33/47) with double mutations harbored compound mutations. In contrast to prevailing stepwise models, we found sequential, branching, and parallel routes to compound mutations (Figure 1A). The frequency of clones harboring compound mutations with >2 missense mutations was low (10%). At the same time, the likelihood of silent mutations increased disproportionately with the total number of mutations per clone, suggesting a limited tolerance for missense mutations in the *BCR-ABL1* kinase domain [4]. Silent mutations are not subject to selection pressure and provide a convenient measure of mutation rate. Their increased frequency in clones with multiple mutations suggests clones that instead acquire additional missense mutations are not competitive, possibly because of impaired kinase function.

In 39% of patients with compound mutations, subclones representing each of the component mutations co-existed with the compound mutations (Figure 1A). This observation dictates that at least one of the two component mutations arose independently in single and compound mutant subclones and opens the possibility that the same mutation detected at two time points might be of different clonal origin. Thus, disease recurrence with the ‘same’ mutation after a transient response does not definitively establish a clonal relationship. This might also explain why a patient with a certain mutation may show a different level of sensitivity to the same TKI during the course of treatment (Figure 1B). Alternatively, a clone may acquire additional non *BCR-ABL1* kinase mutation-based resistance mechanisms.

Given the large number of kinase domain mutations identified in patients with resistance, independent acquisition of the very same mutation is puzzling and strongly suggests a tendency for nonrandom acquisition of certain mutations over others. Various DNA repair mechanisms are defective in CML cells [2], but this does not explain the preferential and repetitive acquisition of identical mutations at certain residues. It will be interesting to examine whether the point mutation rate in other regions of the CML genome is elevated and whether there are recurrent mutational hotspots. Such a study would shed light on whether the *ABL1* kinase domain is located in a part of the genome that is unusually predisposed to mutation acquisition [6]. Cloning and sequencing of the *BCR-ABL1* kinase domain in our study of CML samples also revealed many mutations that were not detected by conventional Sanger direct sequencing [4]. The relatively high frequency of these low-level mutations might be due to DNA errors induced by the BCR-ABL1 oncoprotein in CML cells [3].

Models to explain mutational complexity in cancer have invoked a sequential, orderly hierarchy in which the progression of events can be deduced retrospectively. Our study in CML challenges this simplistic framework [4]. Clinical observations are consistent with acquisition of the second mutation by the pre-existing single mutant clone [5, 8], suggesting that introduction of mutations in *BCR-ABL1* kinase domain happens constantly in certain CML patients [9] and that either: 1) not all observed compound mutations predate TKI therapy or 2) the observed compound mutations predate TKI therapy but existing detection methods are not sufficiently sensitive for identifying low-level mutations in pre-therapeutic samples. The latter, if proven, suggests that mutation acquisition is sharply curtailed on TKI therapy. Our study demonstrates that compound mutations are common in patients with two *BCR-ABL1* mutations and frequently reflect a highly complex clonal network whose evolution may be limited by the negative impact of missense mutations on kinase function.
